# Mex-3 RNA binding MEX3A promotes the proliferation and migration of breast cancer cells via regulating RhoA/ROCK1/LIMK1 signaling pathway

**DOI:** 10.1080/21655979.2021.1964155

**Published:** 2021-09-05

**Authors:** Li Yan, Hongjing Li, Wenbo an, Wei Wei, Xiaolei Zhang, Linlin Wang

**Affiliations:** a Department Of Pathology, Dongying People’s Hospital, Dongying City, Shandong Province, China; b Department Of Radiology, Dongying People’s Hospital, Dongying City, Shandong Province, China; c Department Of Oncology, Dongying People’s Hospital, Dongying City, Shandong Province, China

**Keywords:** MEX3A, RhoA/ROCK1/LIMK1 signaling pathway, breast cancer

## Abstract

Breast cancer has been known as cancer with high mortality rates. It has been studied that MEX3A (Mex-3 RNA Binding Family Member A) is involved in carcinogenesis by accelerating cancer proliferation and migration. Therefore, this research aimed to study how MEX3A regulates the biological behaviors of breast cancer. Firstly, we used GEPIA and KM-plotter databases to evaluate MEX3A expression in human breast cancer tissue compared to adjacent normal tissue. Immunohistochemistry was employed to assess MEX3A protein expression in clinical specimens. MEX3A mRNA expression level was assessed through quantitative real-time PCR (RT-qPCR). Western blotting was used to detect protein expression. Moreover, Cell Count Kit-8 (CCK-8) assay, wound healing assay and transwell invasion assay were used to determine the proliferation, migration and invasion of breast cancer cells, respectively. Our study found that MEX3A expression level was much higher in human breast cancer tissues as compared to adjacent normal tissues. Similarly, breast cancer cell lines showed higher expression of MEX3A as compared to the normal breast cells. This higher expression of MEX3A was linked with the poor survival of breast cancer. Moreover, we found that overexpression of MEX3A stimulated proliferation and migration in the breast cancer cells. However, inhibition of MEX3A significantly reduced the proliferation and migration of breast cancer cells. In addition, we determined that MEX3A could activate RhoA/ROCK1/LIMK1 signaling in the breast cancer cells. Overall, our study concluded that MEX3A promotes its migration and proliferation in breast cancer cells via modulating RhoA/ROCK1/LIMK1 signaling pathway.

## 1. Introduction

Breast cancer made up of 24.2% of all female cancer cases in 2018 and 15% of female cancer deaths were due to breast cancer [1]. According to its pathological characteristics, breast cancer has been categorized into three different subtypes: estrogen receptor (ER)/progesterone receptor (PR) overexpression, epidermal growth factor receptor 2 (HER2) overexpression and triple negative breast cancer (negative for all three receptors) [2–4]. Breast cancer with ER/PR, and HER2 overexpression, as well as triple negative breast cancer accounts for approximately 60, 20 and 20% of the total breast cancer cases, respectively [5]. Each subtype has different prognosis, recurrence rate and treatment strategy [2]. Despite the advances in breast cancer research, the prognosis remains unsatisfactory [6,7]. Multidrug resistance and metastasis are still the main obstacles to breast cancer treatment [8]. Therefore, elucidating the molecular mechanisms of breast cancer initiation and progression is necessary. Mex-3 RNA Binding Family Member A (MEX3A) is in the complementary sequence (156,072,013–156,081,998) with 9986 base pairs [9]. MEX3A is a member of human MEX3 family and is homologous to MEX3B, MEX3C and MEX3D. This family of genes has a highly KH-conserved domain, which can bind RNA and ssRNA and even participates in DNA transcription and mRNA translation [10,11]. Studies have found that MEX3A is involved in the pathogenesis of cancer [12–14]. In pancreatic ductal adenocarcinoma, MEX3A expression is significantly up-regulated which is linked with advanced stage of pancreatic ductal adenocarcinoma. However, the downregulation of MEX3A can inhibit the growth of pancreatic ductal adenocarcinoma *in vivo and in vitro* [12]. MEX3A can also interact with LAMA2 through PI3K/AKT signaling to promote lung adenocarcinoma metastasis [13]. In addition, high MEX3A expression is associated with the advanced stage of malignancy and poor prognosis of triple negative breast cancer. Generally, upregulation of MEX3A promotes proliferation and migration in triple negative breast cancer via modulating PI3K/AKT signaling [14].

However, MEX3A dependent regulation of breast cancer has not been fully elucidated. Therefore, we designed this study to evaluate the expression of MEX3A in human breast cancer tissues and cell lines. Moreover, we also aimed to determine the functions of MEX3A in breast cancer cells. Finally, we assessed the MEX3A dependent regulation of RhoA/ROCK1/LIMK1 signaling pathway involved in the progression of breast cancer.

## 2. Materials and methods

### 2.1. Clinical sample

All participants signed an informed consent form before their operation started. This study was approved by the ethics committee of our hospital, and carried out under the terms of the Helsinki Declaration. Forty-three breast cancer cases from June 2017 to March 2019 in our hospital were selected. We collected samples from mastectomy specimens from women undergoing surgery for breast cancer, and adjacent cancerous tissue specimens were obtained at least 5 cm away from the tumor. All breast cancer patients did not receive any treatment before surgery. All clinical specimens were kept at −196°C in liquid nitrogen until they were used for RNA extraction and other experiments [12].

### 2.2. Cells and cell culture

Four breast cancer cell lines (SKBR3, BT474, MCF-7 and MDA-MB-231) and human breast epithelial cell line (MCF-10A) were bought from BeNa Culture Collection Biological Technology Co. Ltd. (BeNa, China). Cells were cultured in Dulbecco’s modified Eagle’s medium (Invitrogen, Carlsbad, CA, USA) added with 10% fetal bovine serum (Sciencell, LA, USA) and 1% penicillin-streptomycin (100 U/ml penicillin and 100 µg/ml streptomycin, Gibco). Cells were incubated at 37 ^o^C with 5% CO_2_ and 95% humidity [14].

### 2.3. Cell transfection

Small interfering RNA (siRNA) of MEX3A (si-MEX3A) and the negative control siRNA (si-NC), pcDNAMEX3A (MEX3A) and the negative control pcDNA (NC) were synthesized by Genepharma (China). The transfection was facilitated with Lipofectamine® 3000 reagent following the manufacturer’s instructions (Invitrogen, Carlsbad, CA, USA) [13].

### 2.4. Immunohistochemistry

Tissue sections (4 μm) were heated at 60°C for 30 min and next subjected to 3% H_2_O_2_ at 25°C for 10 min. Following that, slides were soaked in distilled water for 10 min, blocked with 10% BSA at 25°C for 30 min, incubated with the primary antibody MEX3A (Abcam, ab79046, 1:100 dilution) overnight. Afterward, tissue sections were rinsed 2 times for 5 min with PBS. Following that, these sections were incubated at for 60 min at 25°C. These tissue sections were added with diaminobenzidine (DAB) and then stained with hematoxylin for 30 s. Next, ethanol was used to dehydrate tissue sections and slides were mounted. Finally, the images of these tissue sections were taken. IHC results were independently scored by two senior pathologists in our hospital [12].

### 2.5. Western blotting

Protein samples were collected from cell lysates. Protein was loaded onto 10% sodium dodecyl sulfate-polyacrylamide gel (SDS-PAGE), and then were transferred to polyvinylidene fluoride (PVDF) membranes (Bio-Rad, Hercules, CA, USA). At room temperature, these membranes were blocked in PBS containing 5% nonfat milk for 1 h, and then incubated with primary antibodies overnight at 4°C. The following were the primary antibodies in this research: anti-MEX3A (dilution: 1:500, Abcam, UK, ab79046), anti-RhoA (dilution: 1: 500, Abcam, UK, ab187027), anti-ROCK1 (dilution: 1:1000, Abcam, UK, ab134181), anti-LIMK1 (dilution: 1:500, Abcam, UK, ab81046), anti-β-actin (dilution: 1:1000, Abcam, UK, ab8226), anti-RhoA (phospho S188) (dilution: 1:1000, Abcam, UK, ab41435), anti-phospho-ROCK1 (Tyr913) (dilution: 1:1000, Invitrogen, USA, PA5-105054) and anti-phospho-LIMK1 (Thr508) (dilution: 1:1000, Invitrogen, USA, PA5-104925). After being washed with TBST, these membranes were incubated with secondary antibody at 25°C for 1 h. Protein blots were visualized using enhanced chemiluminescence (Millipore, USA) [14].

### 2.6. Quantitative real-time PCR (RT-qPCR)

The total RNA was extracted from the cells using TRIzol reagent (Invitrogen, USA). Then, the extracted RNA was reversely transcribed into cDNA using the ReverTra Ace qPCR RT Kit (Toyobo, Japan). RT-qPCR was carried out via SYBR Premix Ex Taq II (RR820A, Takara, Japan). Three independent experiments were carried out to analyze each sample. β-actin was employed as an endogenous control. The specific primer sequences are mentioned in Table 1 [13].

**Table 1.** List of primers used in this study

Gene	Forward Primer	Reverse Primer
MEX3A	CGGAGTGGACTCTGGCTTTGAG	CAGAGGAGAAGAGCACGGAGGT
β-actin	CTTAGTTGCGTTACACCCTTTCTTG	CTGTCACCTTCACCGTTCCAGTTT

### 2.7. Cell proliferation assay

CCK-8 assay (Biotechwell, Shanghai, China) was employed to measure cell proliferation. In short, cells (5 × 10^3^ cells/well) were placed onto 96-well plates. Then, CCK-8 solution (10 µL) was added into each well. Next, the plates were incubated for 2 h at 37°C. The absorbance value was measured at 450 nm with a microplate reader (Thermo Labsystems, Waltham, MA, USA) [14].

### 2.8. Wound healing assay

In short, cells (5 × 10^4^ cells/well) with 80% confluency were plated onto a six-well plate. We next used a sterile plastic micropipette tip to scrape the cells to make an artificial wound. Pictures were taken before the scratch and at 24 h after the scratch was created. Nikon NIS-Element ImagePro Plus 6 was used to measure wound closure [13].

#### 2.9. Transwell assay

Cell suspension was prepared with the culture medium, and the cell density was at 2 × 10^5^ cells/mL. We took 200 μL of cell suspension and inoculated cells in Transwell chambers (8 μm pore size), and 600 μL of the culture medium was placed at the lower chamber. After these chambers were cultured for 36 h at 37°C, the culture medium at the upper chamber was discarded. Next, a cotton swab was employed to carefully wipe cells in the upper layer of the chamber that did not pass through the filter membrane. After the upper chamber was washed with PBS, these membranes were fixed with 4% paraformaldehyde solution for 30 min. Following that, 0.1% crystal violet solution was utilized to stain these membranes for 30 min. Under microscope, cells passing through the membrane were counted in 5 fields, and average value was taken to reflect the migratory property of each group [13].

### 2.10. Statistical analysis

SPSS 22.0 software (spssinc, IL, USA) was utilized for us to analyze our data, and our data were expressed as mean ± standard deviation (SD). Paired t test was used for comparison between the two groups, and one-way analysis of variance was used for multiple groups. P value < 0.05 was considered statistically significant [12].

## 3. Results

### 3.1. MEX3A expression is upregulated in breast cancer tissues and cells

**Figure 1.**
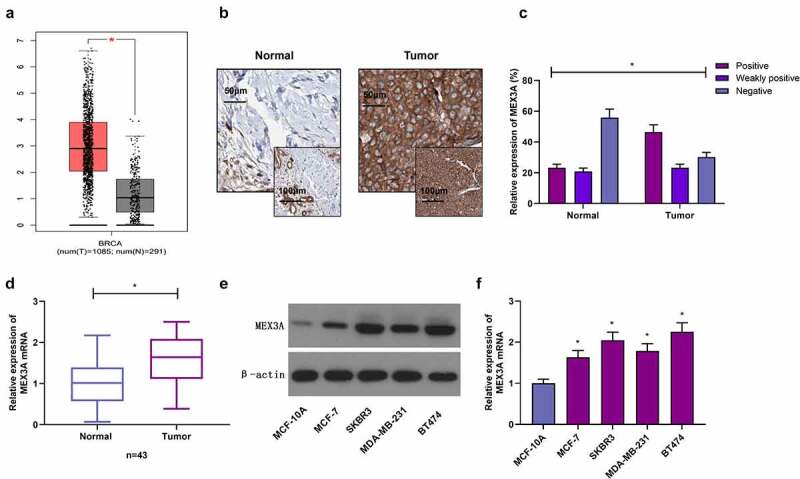
MEX3A expression is increased in breast cancer. (a) MEX3A expression in breast cancer based on GEPIA database. (b) Immunohistochemistry was used to measure MEX3A expression levels in clinical specimens. (c) The strong positive expression rate, weak positive expression rate and negative expression rate of MEX3A in clinical samples. (d) MEX3A mRNA expression level in clinical samples. Western blotting (e) and RT-qPCR (f) were used to measure MEX3A expression in breast cancer cells and MCF-10A cells. *P < 0.05

Expression of MEX3A is associated with the cancer proliferation and migration in several cancers. This study to designed to evaluate the expression of MEX3A in human breast cancer tissues and cell lines. Moreover, we also aimed to determine the functions of MEX3A in breast cancer cells. Finally, we assessed the MEX3A dependent regulation of RhoA/ROCK1/LIMK1 signaling pathway involved in the progression of breast cancer. Through GEPIA database (http://gepia.cancer-pku.cn), MEX3A expression in breast cancer tissues was found to be much higher, in contrast to the control group (Figure 1(a)). Further, immunohistochemistry was utilized to measure MEX3A protein expression in clinical samples. The results showed that MEX3A protein expression in adjacent normal tissues was strongly positive in 23.26% of the total samples, weakly positive in 20.93% of the total samples, and negative in 55.81% of the total samples. In breast cancer tissues, MEX3A protein expression was strongly positive in 46.51% of the total samples, weakly positive in 23.26% of the total samples, and negative in 30.23% of the total samples, suggesting that MEX3A expression in breast cancer may be upregulated (Figure 1(b,c)). Similarly, MEX3A mRNA expression level in breast cancer tissues was significantly higher, in contrast to adjacent normal tissues (Figure 1(d)). Moreover, in contrast to MCF-10A cells, MEX3A expression in breast cancer cells was upregulated (Figure 1(e,f)). These results indicate that MEX3A may regulate breast carcinoma progression.

### 3.2. MEX3A upregulation is associated with poor prognosis in breast cancer

**Figure 2.**
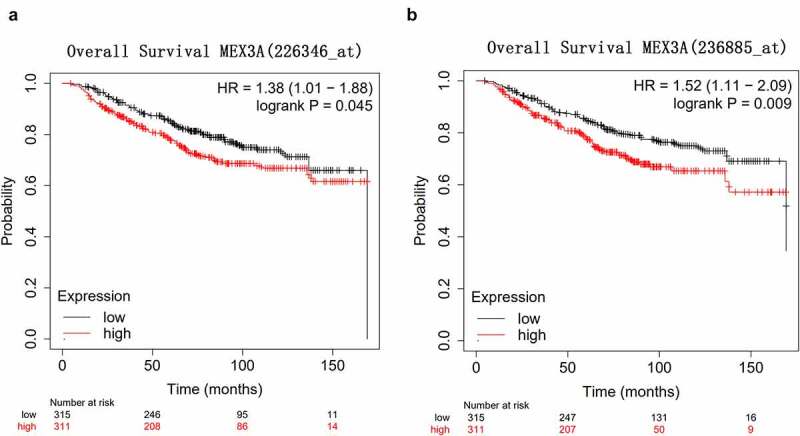
High MEX3A expression is associated with the poor prognosis of breast cancer patients. (a) The relationship between MEX3A (probe: 226346) and the overall survival of breast cancer patients. (b) The relationship between the expression level of MEX3A (probe: 236885) and the overall survival of breast cancer patients

KM-plotter database (http://kmplot.com/analysis/) was utilized to explore the correlation between MEX3A and the prognosis of breast cancer. The overall survival of breast cancer patients with high MEX3A expression was significantly shorter than that of patients with low MEX3A expression (Figure 2(a,b)). This indicates that MEX3A might may function as an oncogene.

### 3.3. MEX3A promotes the proliferation and migration in breast cancer cells

**Figure 3.**
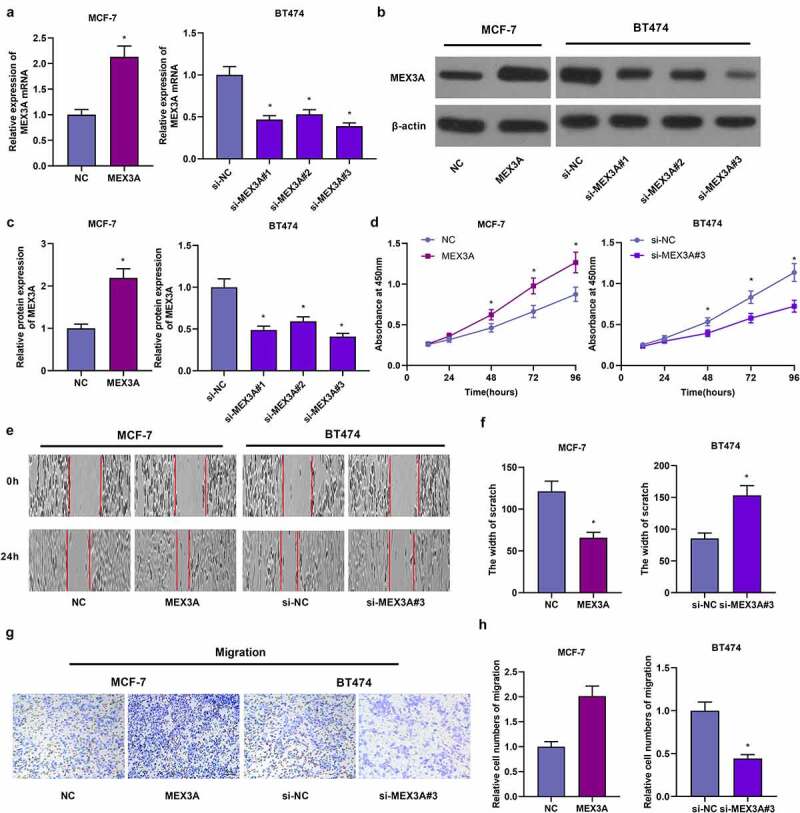
MEX3A promotes the proliferation and migration of breast cancer cells. RT-qPCR (a) and Western blotting (b-c) to measure MEX3A expression levels. (d) CCK-8 assay to evaluate cell proliferation. (e-h) The wound healing assay and Transwell assay to assess cell migration and invasion respectively. *P < 0.05

We transfected MEX3A-overexpressing vectors into MCF-7 cells, and transfected MEX3A siRNAs into BT474 cells and the transfection efficiency was assessed (Figure 3(a-c)). Subsequently, CCK-8 assay was utilized to evaluate cellular proliferative capacity. When compared to the control group, MEX3A overexpression significantly stimulated the proliferation of MCF-7 cells (Figure 3(d)). Yet, in contrast to the control group, knocking down MEX3A reduced the proliferation of BT474 cells (Figure 3(d)). In addition, the overexpression of MEX3A enhanced the migratory property of MCF-7 cells, while the downregulating MEX3A inhibited the migration of BT474 cells (Figure 3(e-h)). Thus, in breast cancer cells, MEX3A can facilitate their proliferation and migration.

### 3.4. MEX3A activates RhoA/ROCK1/LIMK1 signaling in breast cancer cells

**Figure 4.**
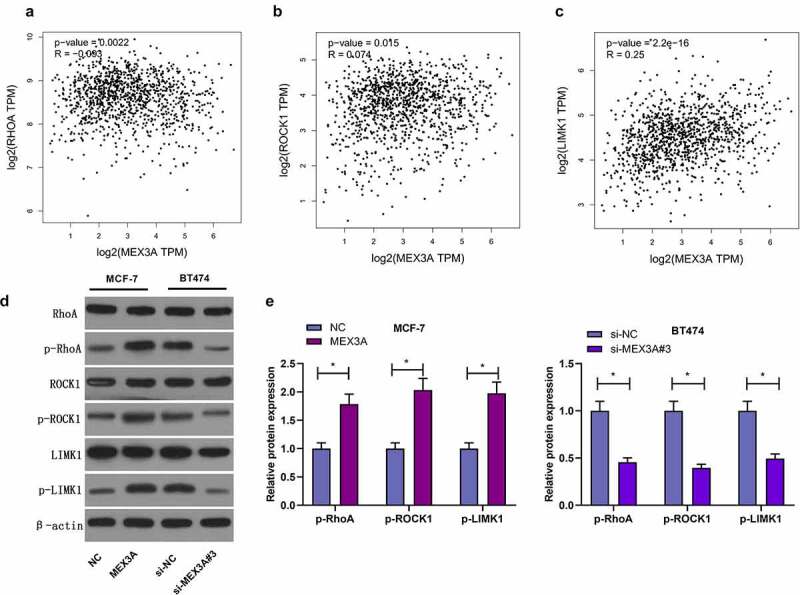
MEX3A activates RhoA/ROCK1/LIMK1 signaling in breast carcinoma cells. (a-c) The relationship between the expression levels of RhoA, ROCK1 and LIMK1 proteins and MEX3A expression level by using GEPIA database. (d and e) The protein expression of p-RhoA, p-ROCK1 and p-LIMK1 in breast cancer cells with MEX3A overexpressed or knocked down. *P < 0.05

It has been well documented that RhoA/ROCK1/LIMK1 signaling pathway regulate several biological processes. Next we used the GEPIA database, and found that the expression levels of RhoA, ROCK1 and LIMK1 were correlated with MEX3A expression level (Figure 4(a-c)). Therefore, we investigated whether MEX3A affected RhoA/ROCK1/LIMK1 signaling in breast cancer cells. MEX3A overexpression enhanced the protein expression levels of p-RhoA, p-ROCK1 and p-LIMK1 in MCF-7 cells and that the knockdown of MEX3A downregulated the expression of p-RhoA, p-ROCK1 and p-LIMK1 (Figure 4(d,e)). In summary, these data indicate that MEX3A can enhance RhoA/ROCK1/LIMK1 signaling in breast cancer cells.

## 4. Discussion

MEX3 is a KH-domain protein, which was first discovered in *Caenorhabditis elegans* (*C. elegans*) [15]. Human MEX3 protein is a type of RNA-binding protein that is highly conserved in evolution. It shares similarity with the MEX3 protein in *C. elegans*. Four MEX3 proteins are mainly concentrated in cellular cytoplasm and can pass through the CRM1 channel between cytoplasm and nucleus. MEX3A gene is one of the four human homologous MEX3 genes, and it is involved in the regulation of mRNA [9,10]. Studies have shown that both MEX3A and MEX3B are the important components of cytoplasmic processing bodies (P bodies) and these tow proteins are mainly involved in mRNA degradation [16,17]. MEX3A is a post-transcriptional regulator, which is reported to regulate the progression of many human malignancies. MEX3A expression is upregulated in hepatocellular carcinoma (HCC) and corelated with the stage of HCC and high MEX3A protein expression level predicts the poor prognosis of HCC patients [18]. RNA-binding ubiquitin ligase MEX3A promotes the progression of glioblastoma by inducing the ubiquitination and degradation of RIG-1 [19]. In gastric cancer, suppressing MEX3A inhibits cellular proliferation and migration [20]. In this study, MEX3A expression in breast cancer is upregulated. In addition, overexpressing MEX3A considerably promotes the proliferation and migration of MCF-7 cells, while MEX3A knockdown has the opposite effect. Thus, MEX3A may promote the carcinogenesis and development of breast cancer.

RhoA is an important member of the Rho family of small G proteins which are expressed in cellular cytoplasm and membrane, and it can regulate the cytoskeleton structure of cells [21]. Rho-related protein kinase (ROCK) is the downstream effector of RhoA, and there are two subtypes: ROCK1 and ROCK2. The Rho/ROCK signaling pathway regulates cellular growth, differentiation, migration and development [22,23]. This signaling pathway is also required for neurite outgrowth, bone formation, back closure, and myogenesis [24]. In addition, the dysregulation of the Rho/ROCK signaling pathway is related to different kinds of diseases, for example, cancer [25]. FPPS can mediate the epithelial-mesenchymal transition (EMT) and invasion of TGF-β1-induced non-small cell lung cancer cells via RhoA/ROCK1 signaling pathway [26]. In prostate cancer, suppressing RhoA/ROCK1 signaling inhibits cellular proliferation and EMT [27]. Studies have shown that the expression of ROCK1 and RhoA are synergistically upregulated in metastatic breast cancer [28]. The increased expression of RhoA or ROCK1 is related to the progression of breast cancer [29,30]. Therefore, MEX3A modulates the activity of RhoA/ROCK1 signaling in breast cancer. Also, the phosphorylation levels of RhoA and ROCK1 decreased with MEX3A knocked sown, and the phosphorylation levels of RhoA and ROCK1 increased after MEX3A overexpression. In addition, the activation of LIMK1 is regulated by MEX3A. This study found that MEX3A overexpression significantly enhanced the phosphorylation level of LIMK1, while the knockdown of MEX3A significantly reduced its phosphorylation level. LIMK1 is another downstream factor of the Rho signaling, which is essential to tumor metastasis [31]. The ROCK/LIMK signaling may convert normal cells into cancer cells [32]. Additionally, studies have shown that IRX5 can negatively regulate RhoA/ROCK1/LIMK1 signaling pathway to promote the metastasis of colorectal cancer cells [33]. Similarly, FHOD3 promotes the occurrence of medulloblastoma by regulating RhoA/ROCK1/LIMK1 signaling [34]. Also, the phosphorylation of RhoA, ROCK1 and LIMK1 in breast cancer cells is positively regulated by MEX3A. Therefore, we speculate that MEX3A promotes the progression of breast cancer through RhoA/ROCK1/LIMK signaling pathway.

## 5. Conclusion

This study demonstrated that MEX3A expression is increased in breast cancer. This upregulation of MEX3A accelerates the proliferation and migration of breast cancer cells. Moreover, inhibition of MEX3A decreased cell proliferation and migration in breast cancer cell lines. Furthermore, MEX3A can regulate RhoA/ROCK1/LIMK signaling pathway in breast cancer cells. Overall, our research indicates that MEX3A and RhoA/ROCK1/LIMK signaling may be new targets for breast cancer treatment.

## Highlights

1. MEX3A expression level increases in human breast cancer tissues and cell lines.
2. Overexpression of MEX3A stimulates proliferation and migration in the breast cancer cells.
3. MEX3A activates RhoA/ROCK1/LIMK1 signaling in the breast cancer cells.
